# The Kock Pouch in Modern Practice Revisited: A Literature Review and Clinically Relevant Summary

**DOI:** 10.3390/jcm14238541

**Published:** 2025-12-02

**Authors:** Rozan Marjiyeh Awwad, Pär Myrelid, Hayim Gilshtein

**Affiliations:** 1 Unit of Colorectal Surgery, Department of General Surgery, Rambam Health Care Campus, Haifa 3109601, Israel; h_gilshtein@rambam.health.gov.il; 2 Department of Biomedical and Clinical Sciences, Linköping University, 581 83 Linköping, Sweden; par.myrelid@liu.se; 3 Department of Surgery in Linköping, Region Östergötland, 582 24 Linköping, Sweden; 4 The Ruth & Bruce Rappaport Faculty of Medicine of the Technion, Israel Institute of Technology, Haifa 3109601, Israel

**Keywords:** Kock pouch, continent ileostomy, IPAA failure, surgical education, review

## Abstract

In patients requiring an ileostomy, the selection of the reconstruction method significantly influences long-term functionality and quality of life. Engaging young patients with the reality of a permanent ileostomy for the remainder of their lives is particularly sensitive and naturally encourages the search for alternative options that may be offered in select cases. Although the end ileostomy and ileal pouch–anal anastomosis (IPAA) continue to be the standard procedures, both possess inherent limitations. When neither approach is feasible, or when these reconstructive techniques have been unsuccessful, having an alternative such as the continent ileostomy (CI) (Kock pouch) proves highly valuable. This review aims to summarize the current indications, contraindications, technical challenges, and outcomes of the Kock pouch to provide surgeons, especially persons with less experience with this procedure, with relevant clinically meaningful information for proper counseling and management of patients.

## 1. Introduction

The Kock pouch, also known as a continent ileostomy, is an internal reservoir created from a segment of small bowel as an alternative to traditional ileostomy and allows voluntary evacuation of fecal material through a nipple valve constructed from the small bowel itself distal to the reservoir. This reconstructive procedure, when performed, aims to replace the standard ileostomy. The CI was first described by Nils Kock in 1969 as a surgical solution for stool continence, following his earlier work in 1964, in which he introduced the concept of an internal reservoir designed originally as a bladder substitute [1].

Initially, the procedure described represented a significant breakthrough for patients, being the only real alternative to a permanent ileostomy; however, its utilization became limited following the introduction of the ileoanal pouch anastomosis (IPAA). The concept of a direct anastomosis of ileum to the anal sphincter complex was first delineated by Rudolph Nissen in 1933, and was later developed into the modern ileal pouch–anal anastomosis (IPAA) technique, which was described in 1978 by Sir Alan Parks and Professor John Nicholls [2]. Later on, in 1980, Utsunomiya et al. introduced the J-Configuration of the pouch. Over time, long-term outcomes indicated a 10–15% incidence of non-functioning IPAA [3], which emerged as a primary factor influencing the selection of the CI. Additional common indications encompass proctocolectomy for inflammatory bowel diseases, particularly in patients with ulcerative colitis, and in highly selective cases of Crohn’s disease. Nonetheless, it has been demonstrated that complication rates are elevated in patients with Crohn’s disease, which has limited the indication for CI for highly selected cases with long-standing colitis [4,5,6,7,8].

Furthermore, the procedure has become a feasible alternative for patients with familial adenomatous polyposis, including those who are contraindicated for IPAA construction due to pelvic disease, prior radiation therapy, low rectal cancer with sphincter involvement, sphincter damage, psychological refusal of ileostomy, perianal diseases, or inability to tolerate the external application of a traditional ileostomy [9].

Another indication reported in cases is slow transit constipation, which supports the use of a CI following proctocolectomy due to benign but chronic colonic disease [5,10,11,12].

Compared to conventional ileostomy, patients with CI reported a better body image free from externally applied material and a higher quality of life [5,8]. With CI, patients complain less about skin irritation caused by bowel content. Physical activities are not hindered. While CI offers lifestyle and psychosocial benefits for motivated patients willing to accept higher long-term reoperation risks, the conventional ileostomy is technically less demanding and has lower complication and reoperation rates. However, younger patients with a long-term prognosed ileostomy benefit from the application of the CI [6,8,9].

The physical principle of the CI can be assumed as a low-pressure maneuver that keeps the stool in the pouch while the intra-pouch pressure remains lower than the constructed valve’s pressure, allows controlled bowel emptying through regular self-catheterization, and offers an alternative to traditional stoma care [13,14]. The reservoir should contain 100–200 mL of fluid, which can be expanded as it becomes trained. Initially, the procedure was described as a hand-sutured method without any valve construction [1]. Later on, it became more reliable and evolved to include the creation of a nipple valve [15] and variations in the shape of the pouch itself with suturing or stapling options [16]. This approach is less frequently adopted. Despite the need for revision surgery, sometimes due to suboptimal technique during the initial procedure but more often due to slippage of the nipple valve and/or detachment of the pouch from the abdominal wall, patients report a high level of satisfaction and quality of life, and many returns to everyday activities even after revision surgery [5,8].

Herein, we describe the technical aspects, contraindications for the procedure, and its important and unique complications that the surgeon needs to familiarize herself/himself with.

## 2. Methods

The literature search was conducted using the electronic databases PubMed, Scopus, Web of Science, and MEDLINE. A comprehensive set of search terms was developed by combining keywords and controlled vocabulary related to “Kock pouch,” “continent ileostomy,” “complications,” “outcomes”, “Quality of life”, “revision”, and “Inflammatory bowel disease”. Boolean operators were used to maximize sensitivity and relevance: (“Kock pouch” OR “continent ileostomy”) and (“complications” OR “outcome” OR “revision” OR “failure”). No restrictions were placed on publication date, considering the description of the development history of the Kock pouch throughout the review, and studies in English and German were included, considering the common publication about the Kock pouch in German-speaking nations. Reference lists of retrieved articles and related citations were also reviewed to identify further eligible studies by snowballing. A structured screening process was applied to all identified articles, beginning with title and abstract review, followed by full-text evaluation. Inclusion and exclusion decisions were finalized after thorough examination of each article’s relevance to the predefined research objectives and the key aspects of interest. Additional studies were incorporated during the review process to address conflicting evidence and to facilitate comparative analysis of study outcomes. The corresponding author was responsible for literature screening, data extraction, and synthesis of the most relevant information and findings included in this review. No restrictions were placed on study design; articles of all types were considered eligible if they provided meaningful data related to the research question. Eligible study designs included clinical case reports, prospective and retrospective cohort studies, and systematic reviews. All co-authors carefully reviewed the manuscript, contributed critical revisions, and suggested additional relevant studies for inclusion to strengthen the overall quality and balance of the review.

## 3. Study Design

The review process was initiated by establishing a structured outline and identifying essential domains relevant to the topic. Key thematic domains were predefined to ensure systematic coverage of all clinically relevant aspects. These domains encompassed the historical evolution of the CI and its surgical rationale; technical principles underlying pouch construction; established indications and contraindications; anatomical and technical modifications of the procedure; perioperative and postoperative management strategies; early and late complication profiles; and the integration of the CI into contemporary surgical training, clinical decision-making, and practice. This thematic approach allowed a structured and comprehensive comparison of surgical techniques, clinical outcomes, and reported experiences across different centers.

## 4. The Anatomical Configuration of the Continent Ileostomy

The CI is anatomically divided into four major parts: the afferent loop, the reservoir, the valve, and the efferent loop, which is the stoma site.

The afferent loop should typically constitute a viable, non-inflamed segment of the small intestine. The length of the small intestine must be adequate to ensure effective nutrient absorption. Additionally, the small bowel should lie in an iso-mesenteric rotation.

**Figure 1:**
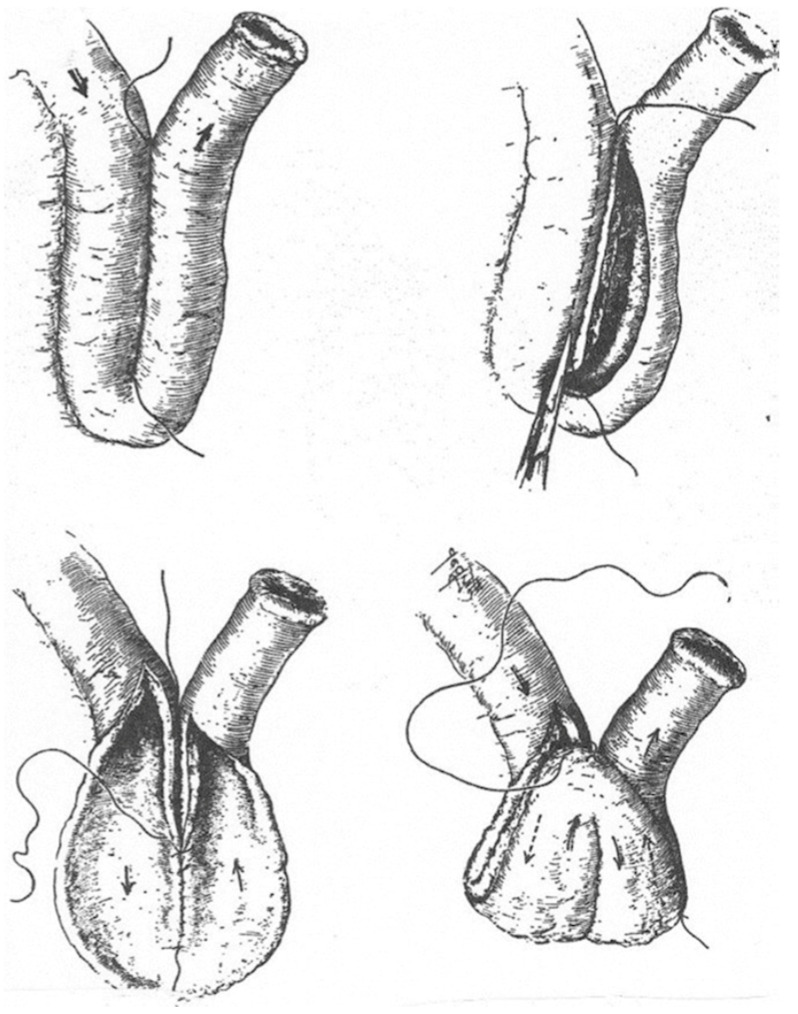
K-Pouch. With permission from Leif Hultén.

There are various methods for building a pouch. The original Kock pouch (K) includes a double folding of the small bowel, which creates a low-pressure pouch (Figure 1).

**Chart 1:**
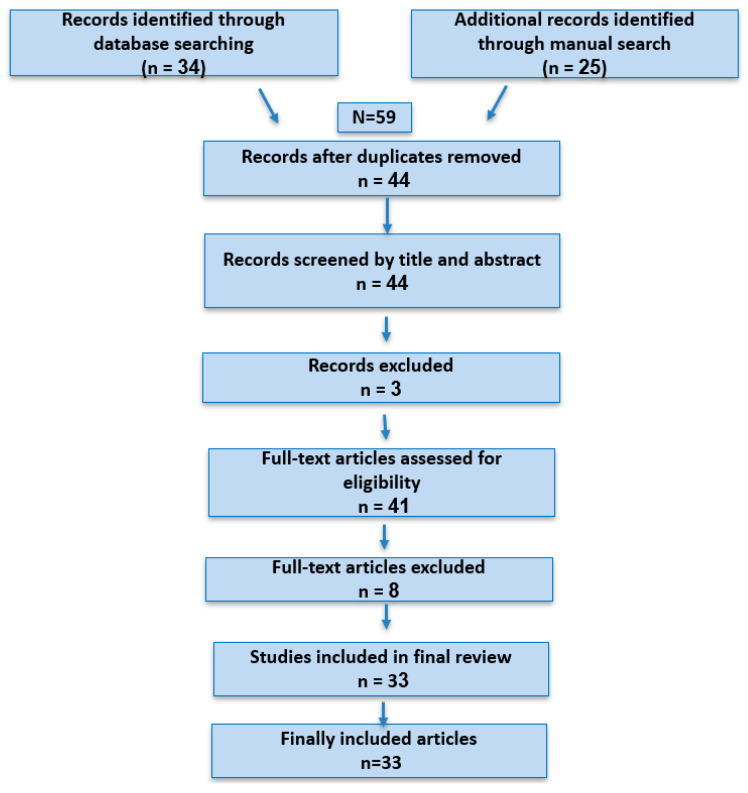
Simplified PRISMA Flow diagram.

Variations in the original pouch—K, S, W, or T—describe the construction of the entero-enteric anastomosis between the loops of small bowel [17,18,19]. Usually, the reservoir requires a small bowel segment of about 30–50 cm, constructed either by hand sewing or stapling. The various forms will be discussed in the following sections (Chart 1).

## 5. Indications and Contraindications

The indications for a CI (in its various forms) are generally fewer than its contraindications. The most common patients to benefit from CI are those with ulcerative colitis or FAP who cannot undergo an ileal pouch–anal anastomosis (IPAA) and have undergone proctocolectomy without the option of preserving the natural defecation pathway or have had a complicated non-functioning IPAA. Additionally, some patients either cannot have or are not interested in IPAA or ileostomy, usually due to incontinence or a malfunctioning sphincter, and sometimes even due to idiopathic incontinence [2,7]. Another group includes those with extensive perianal abscess or fistulization disease, which makes them unsuitable for IPAA. The literature also describes more selective indications, such as proctocolectomy for slow transit constipation. Generally, candidates for this procedure are those requiring ileostomy without small bowel pathology or fistulization potential, and who have the mental and psychological capacity to understand how it works and how to manage it, as well as those who might need revision procedures. Even in consideration of the possibility that patients may require revision procedures over time, the continent ileostomy remains a justifiable and advantageous surgical option, as it continues to offer favorable functional outcomes and sustained improvements in quality of life [1,2,7,10,13,19,20,21,22].

While it is essential to be confident about the indication, it is equally important to have a thorough understanding of the contraindications associated with it. Recognizing these contraindications is crucial for ensuring safety and effectiveness in treatment. These contraindications can be categorized into three distinct groups, each requiring careful consideration and knowledge to appropriately manage and avoid potential risks (Table 1).

1. Patient-related factors to consider include obesity, primarily caused by excessive mesenteric and abdominal wall fat, which lead to technical and anatomical challenges, especially during the construction of the valve and the external stoma site [23]. However, no specific Body Mass Index has been identified as a definitive contraindication for a CI, and this remains a topic of discussion. Nonetheless, poor nutritional status and smoking are well-documented and proven risk factors for insufficiency, leakage, and surgical site infection. Additionally, the presence of desmoid tumors in patients with FAP can complicate or prevent the construction and maintenance of the pouch. These are considered some of the most significant contraindications for performing a continent ileostomy.
2. Anatomical and physiological factors: Insufficient small bowel length can cause short bowel syndrome, impairing nutrient absorption unless compensated for. The optimal small bowel length has not yet been established, but approximately 30 cm should be reserved for pouch creation, including a valve and efferent loop. Psychological barriers that hinder self-catheterization may also be an absolute contraindication. In Crohn’s disease patients, small bowel involvement is an absolute contraindication due to risks of inflammation, leakage, and fistula formation. However, the late onset of small bowel Crohn’s disease in a patient with a pre-existing continent ileostomy does not alone justify removal; medical therapy remains appropriate. The decision depends more on the severity of complications, such as fistulas, especially in the presence of small bowel Crohn’s disease.
3. Comorbidities: Conditions such as portal hypertension, active malignancies, or prolonged steroid use are regarded as absolute contraindications. While portal hypertension elevates the risk of bleeding from varices during any form of surgical procedure, it also markedly increases morbidity and mortality rates. Patients presenting with these conditions may already be at a heightened risk for the initial surgical intervention. Parastomal varices bleeding may necessitate transfusions, complicate endoscopic management of bleeding, and pose a threat of life-threatening hemorrhage [8,24]. Regarding steroid use, the risk of infection is, as generally discussed in the literature, higher and can impair anastomotic healing, increase the rate of postoperative surgical site infections, and raise the leakage rate. Furthermore, patients with active malignancy are considered to have limited life expectancy or could still be prepared for cytotoxic treatment, which also makes it an absolute contraindication for CI.

## 6. The Construction of the Reservoir

The construction of the reservoir and the valve within the pouch has been well described in various publications [9,14,16,17,19]. The method of creating a reservoir can vary depending on the surgeon’s preference and the initial approach. The original Kock pouch, developed by Nils Kock in 1969 [1], was designed with a spherical configuration. After the introduction of the ileal pouch–anal anastomosis (IPAA) by Parks and Nicholls in 1978 [2], both patients and surgeons generally preferred this option over continent ileostomy. However, for patients who cannot preserve their IPAA but still wish to maintain continence, a continent ileostomy remains a viable alternative. In particular, converting a J-pouch into a K-pouch has been considered an effective option in such cases. The various types of reservoirs are described as S-, K-, and T-pouches. All these pouch procedures are performed as a laparotomy. There is still no description of a minimally invasive technique for performing a CI.

### 6.1. The K-Pouch

**Figure 2:**
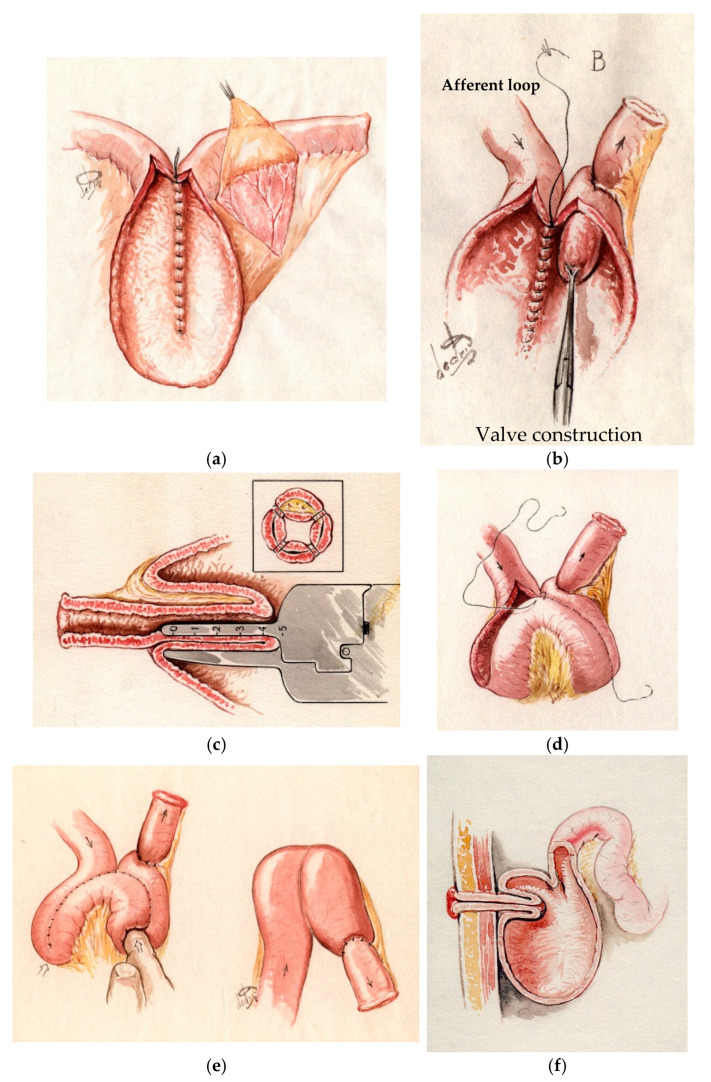
(**a**–**f**): K-Pouch. (**a**) A 30 cm long anti-mesenteric incision with closure of the back wall and excision of the peritoneum on both sides of the mesentery. (**b**) Intussusception of the nipple valve. (**c**) Stapling of the nipple valve. (**d**) Closure of the anterior part of the pouch. (**e**) Creation of a spherical pouch by pushing both ends of the pouch through the mesentery (**f**) Fixation of the pouch to the abdominal wall. With permission from Leif Hultén.

Take a 30 cm segment of the small bowel loop, mark the proximal and distal ends, and perform an anti-mesenteric incision, extending the proximal end of the marked bowel [9]. To incise the bowel, a suction tip can be used as a guide while inserting it into the bowel. Subsequently, a posterior wall suture is performed; alternatively, stapling of the posterior wall may be employed, followed by an incision on the anti-mesenteric side of the bowel. The next step involves removing the peritoneal covering of the mesentery on both sides of the proximal end of the outlet site. This segment of the bowel will serve as the nipple valve. This step facilitates adhesion formation following invaginations. The subsequent phase is creating the valve via an intussusception maneuver of the outlet loop, beginning from the last third of the free loop and telescoping the end of the outlet loop toward the pouch. The nipple length should be approximately 5–6 cm. The next critical step is to fixate the nipple valve at the pouch wall using a knifeless 60 mm stapler. Whether GIA or TA is used depends on the preference of the operator. To consider if the gap that resolves by using the TA should be oversewn. The nipple valve should be fixated in 3 to 4 points, sparing the mesentery of the loop to prevent ischemic damage to the valve (Figure 1 and Figure 2).

Subsequently, the anterior wall should be sutured, ensuring an appropriate distance between the inlet and the nipple valve. This can be accomplished by making the primary antimesenteric bowel incision asymmetrical, extending it towards the proximal loop (inlet side). The next step involves reattaching the base of the nipple to the pouch wall from the external side.

Finally, both ends of the pouch should be guided towards the mesentery, resulting in a more spherical appearance after initially being banana-shaped. After this step, the pouch should have an inlet on the left side and an outlet loop on the opposite side.

The procedure for establishing the stoma site commences by securing the nipple valve to the abdominal wall using a total of eight sutures, avoiding the mesenteric region. The lateral anchoring of the nipple cuff to the anterior sheath of the rectus muscle is achieved with at least two sutures. The remaining sutures serve to anchor the cuff of the nipple to the posterior rectus sheath [9].

### 6.2. The S-Pouch

**Figure 3:**
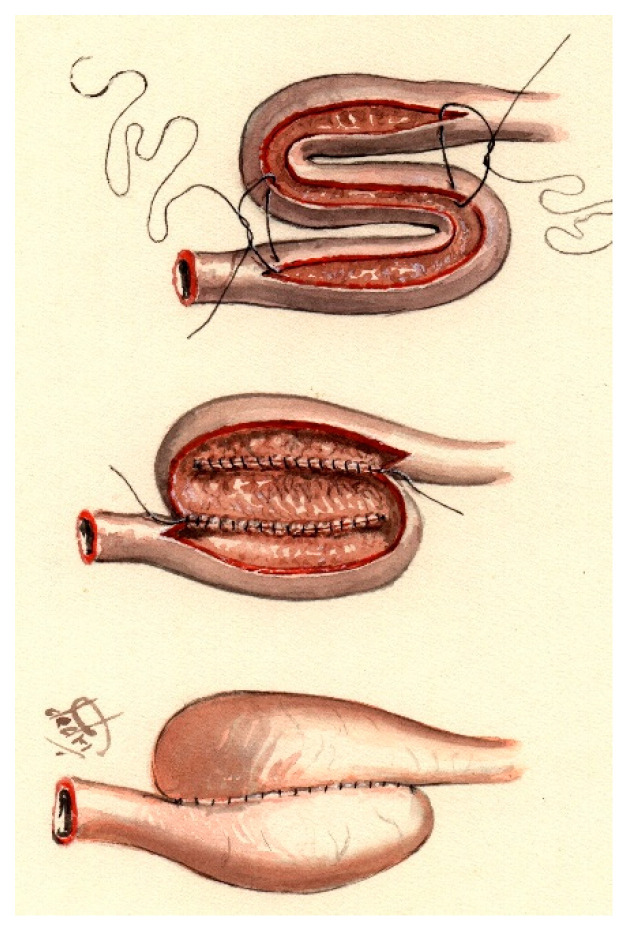
S-Pouch. Folding and suturing of an S-pouch. With permission from Leif Hultén.

Compared to the previously described K-pouch, the S-pouch is constructed using three limbs, which creates increased capacity. The posterior wall will be sutured or stapled, and then the valve will be performed similarly to the previously mentioned method after triangular-shaped deperitonealizing of the mesentery at the valve limb. Some surgeons fixate the valve to the pouch wall either with two knife-less staplings at 4 and 8 o’clock or three firings at 12, 4, and 8 o’clock, while the mesentery lies at 6 o’clock [14] (Figure 3).

### 6.3. The T-Pouch

A further method for constructing the reservoir is the described T-pouch [17,25]. It was introduced in 2002 by Kaiser et al. [16] as a new valve design for the continent ileostomy without performing invagination, aiming to address the high rate of valve dysfunction and revisions seen with the standard CI. This involves constructing a pouch reservoir from two apposed bowel limbs, creating a serosa-lined tunnel at their intersection. The terminal ileum segment (for the valve) is embedded within the serosa-lined tunnel to form the antireflux mechanism, which also helps prevent intussusception [26]. The T-pouch valve is created without intussuscepting the ileum; instead, the terminal ileum is isolated with its blood supply and embedded in a serosa-lined tunnel formed by two apposed bowel limbs that become the reservoir. The reservoir is then fashioned with two ileum limbs. This design is thought to be less prone to slippage and maintains better vascularity of the valve wall. In the small series of six T-pouch cases described by Kaiser et al. [16], three patients experienced complications within 2–18 months, and only one required revision for valve trauma; all remained continent and free of major complications. However, long-term results are still lacking, and the superiority of this technique cannot yet be assumed [16].

### 6.4. Conversion of an Ileal Pouch–Anal Anastomosis (IPAA) into CI

**Figure 4:**
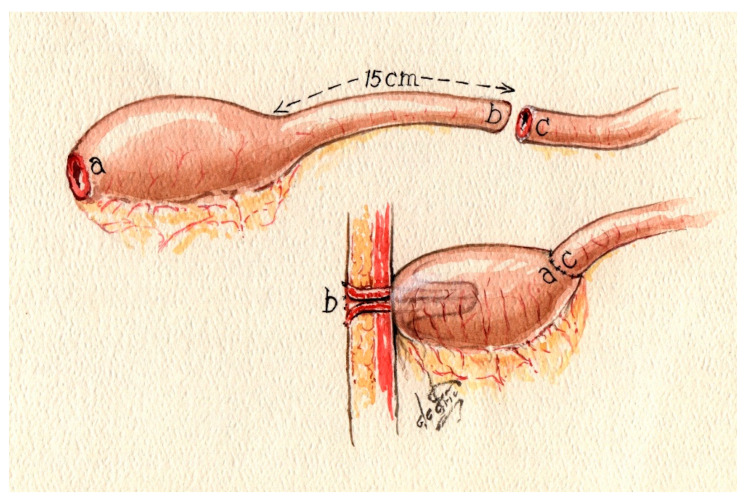
Creation of continent pouch from a previous pelvic pouch. (a) shows the disconnected IPAA (b) distal site of the afferent loop of the IPAA (c) proximal site of the devided afferent loop. Dividing the small bowel 15 cm proximally of the pouch, which will later become the nipple valve. With permission from Leif Hultén.

Since its inception, the IPAA has been acknowledged as one of the most acceptable pouch configurations that preserves the natural pathways for stool evacuation. Introduced in the 1980s, the IPAA has markedly decreased the frequency of CI surgeries across all its variants [13] (Figure 4).

Indication for Conversion: Subsequent long-term outcomes of IPAA procedures have indicated potential complications, some of which have necessitated pouch removal and the formation of an end ileostomy. Patients without contraindications may benefit from converting an existing IPAA into a CI, thereby conserving more of the small bowel length [27,28].

Contraindications: However, patients with small bowel inflammation, severely impaired small bowel function, or a shortened bowel length are not suitable candidates for this conversion [25].

Technical strategies: Ecker et al. [28] described various types of conversion: Type 1 involves the J-pouch conversion without pouch reconstruction but with nipple valve formation, utilizing either the afferent loop of the pouch or the proximal part of the small bowel; Type 2 involves partial pouch reconstruction using either the terminal ileum or the proximal small bowel; and Type 3 comprises a complete reconstruction of the pouch using the proximal ileum or jejunum.

Complications: The postoperative complication rate for this procedure generally ranges from 53% to 56% [25,28,29] and can be divided into early and late complications, as well as pelvic dissection-related versus pouch (including nipple) related complications, based on timing.

## 7. Postoperative Management

**Chart 2:**
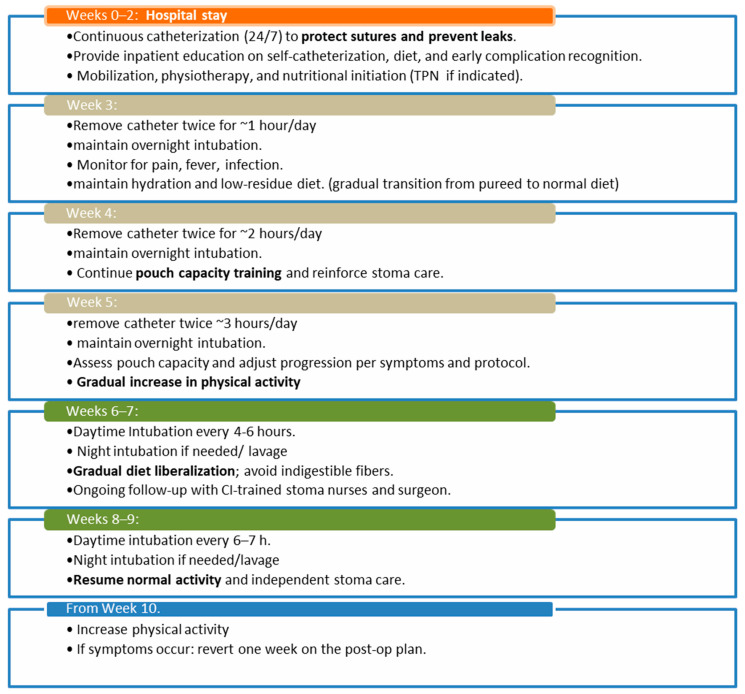
Adapted from Dörner et al., 2018 [27]—Structured 9-week postoperative pouch training program.

The postoperative hospital recovery after the initial procedure lasts about 14 days for newly formed CIs. The total postoperative recovery period may last up to 9 weeks. A 9-week postoperative catheter management plan was described by Doerner et al. in 2018 [27]. The stepwise postoperative catheter-training and dietary advancement strategy presented herein was adapted from the standardized postoperative program proposed by Dörner et al. [27], which provides a systematic approach to pouch maturation following CI surgery (Chart 2).

Initially, the pouch should be continuously catheterized for two weeks. This approach supports healing of sutures and reduces the risk of insufficiency. During this period, patients should be instructed on diet management, self-catheterization, and how to identify problems with a CI. This phase includes mobilization, physiotherapy training, and nutritional initiation. Depending on the patient’s recovery speed and institutional protocols, the pouch should initially be catheterized continuously for two weeks. After that, for 3 weeks, the catheter can be removed for one hour each week, but it will remain in place overnight.

Afterward, the pouch can be gradually extubated for longer periods to gradually increase its volume capacity. The diet should be resumed as soon as possible; patients with malnutrition should not undergo the procedure until their nutritional status improves to lower postoperative complication rates. If necessary, total parenteral nutrition (TPN) can be started simultaneously, especially in patients who develop symptomatic ileus. Stoma nurses, trained for continent ileostomies, are a vital part of the team. Gradual physical reintegration should also be considered. Patients need to be aware of the importance of guidance to avoid excessive fatigue or muscle overwork, particularly of the abdominal wall muscles.

After discharge, the patient should take care of the CI daily without any exceptions. Patients should be aware of the risks associated with neglecting pouch intubation and changing dietary habits, which can lead to complications [30].

In patients with a CI who require intensive care and become unable to perform self-catheterization, there is a significant risk of iatrogenic morbidity. In these cases, continuous decompression of the pouch must be maintained by the responsible medical and nursing staff as a crucial part of peri-intubation and critical care management. Failure to keep the drainage flowing may lead to progressive pouch distension, mechanical bowel obstruction, overflow incontinence, and other serious or potentially life-threatening complications.

Identifying emergencies is one of the most crucial parts of patient education. Situations like pouch obstruction, intubation difficulties, overflow incontinence, or intestinal blockage from previous abdominal surgeries need to be recognized quickly and diagnosed accurately. This responsibility lies with the surgeon—even those who do not routinely perform CI procedures—to prevent unnecessary surgeries and minimize the risk of harming the pouch. Endoscopy can be a helpful diagnostic tool; however, it must be performed with extreme care and only after a thorough understanding of the pouch’s anatomy. Often, a contrast-enhanced CT scan is a safe and very informative initial investigation.

## 8. Complications

Postoperative complications can be categorized into early and late complications.

Early complications encompass common surgical issues and specific complications. These include the following:

Nipple valve complications, such as ischemia, incontinence, and perforation, typically occur from stapling failure or an inadequate suture line. The stapling line of the valve during invagination or during fixation to the pouch wall may impair blood flow, leading to ischemia, which can cause air or stool leakage or even perforation. The diagnosis of this complication is crucial for early surgical intervention.

Pouch-associated complications, including insufficiency and ischemia. The prevention of this complication begins with continuous postoperative drainage of the pouch. After four weeks, the catheter could be removed and the patient should be observed for any abnormal infection signs, such as abdominal pain, fever, or surgical site infection (late).

Surgical site infections: No higher incidence compared to other laparotomies [22].

Intra-abdominal infections, such as abscesses and peritonitis.

Late complications include complications that can appear after 14 days or many years after the index operation.

Nipple and valve complications include prolapse, slippage, stricture, fistulization, or wall defect. Valve dysfunction remains the primary long-term issue and reason for revision [5,7,8]. Patients often report intubation problems and/or incontinence, usually due to poor fixation of the valve to the pouch wall or the pouch to the abdominal wall. Valve slippage can lead to intestinal obstruction, but it seldom requires emergency surgery. Instead, it is crucial to have the availability and the ability to perform endoscopy and adapt intubation, sometimes using the Seldinger technique [31]. Diverse valve designs have been described aiming to reduce the rate of valve dysfunction, like the above-mentioned T-pouch. However, further long-term studies are still needed [17]. Even very late nipple-valve failure should not be regarded as an automatic indication for pouch excision. In appropriately selected and motivated patients, revision surgery performed in experienced centers offers a high likelihood of preserving the continent ileostomy while maintaining satisfactory long-term functional outcomes [20].

Pouch-associated complications include pouchitis and fistulizations. Pouchitis [4] is a common cause of incontinence. The precise etiology remains unclear; however, antibiotic therapy can usually resolve the issue without necessitating further intervention. Nevertheless, it could be a reason for pouch excision. Additionally, the appearance of de novo Crohn’s disease of the pouch has also been described [32].

Intra-abdominal adhesions leading to intestinal obstruction with no evidence of a higher rate compared to laparotomies due to other etiology.

Functional deficiency—incontinence of unknown cause. In these cases, one should perform imaging studies and functional studies and examine anamnestic details to check if the patient is dealing correctly with nutritional restriction and catheterization before concluding that the valve is not functioning.

Outlet obstruction caused by indigestible food necessitates exercising caution regarding dietary practices, as it is essential to prevent non-digestible fibers from becoming lodged in the pouch, which could impede their removal via catheter. This diagnosis should always be considered in CI patients prior to making decisions about surgical intervention. Pouchoscopy remains the standard of care for diagnosing the issue and removing undigestible particles.

Fistulization: Usually, in patients with Crohn’s disease, this leads to incontinence with symptoms. Fistulas can be observed between the pouch wall and the skin, called pouchocutaneous fistulas, or between the valve and the perivalvular skin, which appears as persistent spillage from the perivalvular skin. Pouchoscopy with careful observation is the standard of care. CT fistulography or MRE can also help define the fistula tract sometimes.

Dislocation or volvulus of the pouch can occur due to inadequate fixation to the abdominal wall [24]. Volvulus along the mesenteric axis can cause necrosis and requires immediate surgical intervention.

Cosmetic results: Cosmetic outcomes after continent ileostomy (CI)—though less frequently discussed—are often suboptimal due to the need for laparotomy and frequent revisions [6,16]; yet, most patients still report high satisfaction, likely prioritizing continence and lifestyle gains over abdominal appearance [5,8] (Table 2).

## 9. Discussion

The continent ileostomy initially attracted considerable attention but gradually declined in use with the widespread adoption of IPAA. This procedure is technically demanding and clinically challenging, with the need to identify indications and contraindications for its implementation accurately. Future success depends on setting realistic patient expectations, ensuring acceptance of the relatively high revision rate mentioned in the literature [6,8,25], and preparing patients for the daily management of the ileostomy. Prompt recognition of emergencies is essential.

Performing a continent ileostomy should not be a standard requirement for every general surgeon, nor even for a colorectal surgeon. However, it is essential for a surgeon to understand the essentials of the procedure and how to handle emergency cases until a surgeon specialized in CI can take over the case.

The learning curve for this procedure is considerable. While no fixed number of cases has been established to define surgical proficiency in this field, mentorship constitutes the cornerstone of skill acquisition. Initial procedures should always be performed under direct supervision to ensure the use of accurate techniques, performance, and optimization of patient outcomes.

It is also worth noting that, despite the rare nature of this procedure, patients are usually extremely grateful and satisfied, as it results in a tremendous improvement in their quality of life [2]. Due to the limited number of appropriate cases, it is crucial for surgeons to learn the necessary skills from experienced surgeons and to attend specialized continuing education programs and hands-on courses.

There is currently no official certification for centers performing continent ileostomy in Europe; nonetheless, several institutions are well recognized for their expertise in this field. In Germany, for example, four to five centers are actively engaged in performing this procedure, and some of them offer opportunities to gain deeper insight into the operative technique. In Sweden, the government has decided that only two hospitals (with national responsibilities) will be involved in the care of patients with CI, for both established and new patients. In order to gain proficiency in this procedure, opportunities can still be arranged through personal contact with experienced centers, while no formal fellowship is available at this point.

Recent publications have documented relatively high revision rates in patients with a CI, often derived from meta-analyses aggregating data from multiple studies [8,33]. It is important to note, however, that the surgical technique for this highly complex procedure varies significantly among surgeons. Specialized centers, which see a greater number of suitable patients and perform the operation with higher frequency, generally achieve more consistent outcomes; however, this hypothesis requires further validation [31,33]. Conversely, when the procedure is performed infrequently, the risk of complications tends to increase, analogous to other technically demanding surgeries. Consequently, conclusions regarding complication rates should be approached with caution, as they remain subject to both technical variability and patient selection bias.

## 10. Conclusions

Successful continent ileostomy construction with optimal long-term outcomes depends on a well-justified indication, the use of a proper surgical technique at high-volume specialized centers, prompt recognition of postoperative complications, and comprehensive pre- and postoperative patient education.

## 11. Practical Key Points for Clinicians

1. Continent ileostomy, in all its forms, is a technically demanding procedure with limited indications and numerous contraindications, requiring careful patient selection and specialized expertise.
2. Patients should be aware of the procedure, the possible complications, and the high revision rate, along with the quality of life they may gain with the CI.
3. Careful patient selection is paramount for continent ileostomy (CI). Ideal candidates are those with ulcerative colitis or FAP who cannot undergo IPAA, have adequate small bowel length, and possess the cognitive ability to manage the pouch; contraindications—such as obesity, poor nutritional status, Crohn’s disease, active malignancy, or steroid dependence—must be rigorously excluded to ensure safety and long-term function.
4. The design of the continent ileostomy construction varies with surgeon preference, encompassing K-, S-, or T-pouch configurations; while originally developed as an open procedure, no minimally invasive technique has yet been established.
5. The postoperative management of continent ileostomy requires a structured, stepwise catheter training and dietary advancement plan lasting up to nine weeks, with continuous pouch drainage during the first two weeks to ensure healing. Ongoing patient education, multidisciplinary support (stoma nurse, surgical follow-up, and nutritional consultation), and prompt recognition of pouch-related emergencies are essential to prevent complications and ensure long-term function.
6. Postoperative complications after continent ileostomy are divided into early (e.g., nipple valve ischemia, pouch insufficiency, or infections) and late (e.g., valve slippage, pouchitis, fistulas, or outlet obstruction) events. Early recognition, meticulous surgical technique, and structured follow-up with endoscopic and functional assessment are essential to preserve pouch function and reduce revision rates.
7. As surgery in non-experienced hands carries a high risk of pouch loss, non-urgent cases should be managed conservatively until expert consultation is obtained.
8. Continent ileostomy is a technically demanding procedure requiring precise patient selection, realistic expectation setting, and prompt recognition of complications. Given the steep learning curve, it should be performed only under expert supervision, ideally in specialized centers, with surgeons encouraged to seek mentorship and dedicated training.

## Figures and Tables

**Table 1:** Contraindications for continent ileostomy.

Contraindications for Continent Ileostomy		
	Relative	Absolute
**Patient-related factors**	Obesity	Poor nutritional status
	Desmoid tumors	Inability to maintain catheterization
	Smoking	Insufficient small bowel length
	Multiple abdominal surgeries with a hostile abdomen	
**Anatomical and physiological factors**	Cardiopulmonary disease	Psychological barriers: autism, dementia, etc.
	Short mesentery	Uncontrolled sepsis
		Small bowel involvement in Crohn’s disease
	Prolonged/ongoing steroid use	Portal hypertension
**Comorbidities**	Immunosuppression	Active malignancy
		Cytotoxic therapy

**Table 2:** Postoperative complications.

Complication	Prevalence (%)	Early Complication	Late Complicaitons	Management
**Total complications**	60.40%	36%	73%	Imaging studies, hospitalization, endoscopy or reoperation
**Reoperation rate**	22–66%			
**30-days Mortality**	0–3.6%			
**Nipple valve complications**	40.50%	ischemia, incontinence, and perforation	Incontinence, Slippage, Dysfunction	Pouchoscopy, CT-scan, Reoperation
**Difficult intubation**	13.10%	incompatible fixation	Valve Slippage, Weigh gain, fascial relaxation	intubation under revision (Pouchoscopy)m reoperation for refixation or reconstruction
**Pouch leak/fistula**	16.80%	preoperative malnutrition, intraabdominal sepsis, ischemia of the suture line, insuitable continuos intubation of the Pouch	Usually Fistula as late complication	Pouchoscopy with blute injection, CT- or MRE or fistulography
**Pouchitis**	13%		usually as long term complication	Endoscopy for diagnosing and excluding of crohns’s associated Pouchitis, ensuring the application of the indicated evacuation, Antibiotics, probiotics, topical therapy with steroids or antibiotical irrigations, pouch Excision (1.3–4%)
**Abscess**	11.90%	due to leakage	Contain leakage or perforation	Diagnosing depends on clinical presentation: CT scan, antibiotics, reoperation
**Hernia**	8.60%		Usually as a long term complication	if no symptoms- no intervention needed. Otherwise hernioplasty
**intraabsominal adhesions**	unknown	consider technical failure	no higher incidence than other laparotomies	depends on clinical presentation
**Dislocation or Volvulus of the Pouch**	not reoprted		Inadequate fixation to the abdominal wall	Pouchoscopy, CT scan—Reoperation [23]
**Outlet obstruction**	not reoprted	Valve slippage	Stoma site overriding polyp, valve slippage, undigestive food particles, increased BMI	Pouchoscopy with cautionly intubation using seldinger technique, reoperation, dietry modification

## Data Availability

No new data were created during the writing of this paper.

## Abbreviations

The following abbreviations are used in this manuscript:

KP	Kock pouch
CI	Continent ileostomy
TPN	Total parenteral nutrition
CT	Computer tomography
MRI	Magnetic resonance imaging
FAP	Familial adenomatous polyposis
IPAA	Ileal pouch–anal anastomosis
